# Is a hypothetical melanoma-like neuromelanin the underlying factor essential for the aetiopathogenesis and clinical manifestations of multiple sclerosis?

**DOI:** 10.1186/1471-2377-13-91

**Published:** 2013-07-18

**Authors:** Bernd Krone, John M Grange

**Affiliations:** 1 Institute of Virology, University of Göttingen, Kreuzbergring 57, 37075 Göttingen, Germany; 2 Medical Laboratory, Kurt Reuber-Haus, Herkulesstraße 34a, 34119 Kassel, Germany; 3 London Clinic Cancer Centre B2, 22 Devonshire Place, London W1G 6JA, UK

**Keywords:** Multiple sclerosis, Risk factors, Latency, Melanoma, Neuro-melanin, Epstein-Barr virus, Human endogenous retrovirus, Vitamin D, Geomagnetic disturbances

## Abstract

**Background:**

Multiple sclerosis (MS) has undergone a significant increase in incidence in the industrialised nations over the last 130 years. Changing environmental factors, possibly infections or a lack of or altered timing of them, determine the prevalence of the disease. Although a plethora of aetiological factors, clearly evident in a group of children with MS, appear relevant, there may nevertheless be a single factor essential for the aetiopathogenesis and clinical manifestation of MS.

**Description and discussion:**

This hitherto unknown factor is postulated to be a ‘melanoma-like neuromelanin’ (MLN) dependent on the activation of a gene for syncytin-1. An involvement of MLN could explain the diverse findings in the epidemiology, immunology and pathology of MS, requiring a consideration of a complex infectious background, the human leucocyte antigens, as well as cosmic radiation causing geomagnetic disturbances, vitamin D deficiency, smoking, and lower levels of uric acid.

**Summary:**

In principle, the MLN-based concept is a unifying one, capable of explaining a number of characteristics of the disease. To date, MLN has not been addressed in studies on MS and future work will need to be done on human patients, as there is little or no neuromelanin (the precursor of MLN) in the animals used as experimental models in the study of MS.

## Background

The recent paper by Sajedi and Abdollahi in this journal [1] on geomagnetic disturbances as a risk factor for multiple sclerosis (MS) emphasises the complexity of the pathogenesis of this disease but also encourages a search for an underlying factor that could be affected by such disturbances and other risk factors. A recent broad-ranging epidemiologic study also focuses attention on the aetiology of multiple sclerosis and facilitates a reappraisal of the putative risk factors [2]. The outstanding question is whether, among the plethora of described possible aetiological factors for MS, there is a single, hitherto undescribed, risk factor that is essential for the entire course of the disease process from the initiating events to clinical manifestation.

### Aim of the debate

In response to this issue, we emphasise that the complex epidemiology does indeed point to a plethora of concomitant factors in MS. Moreover, further enigmas, in particular the wide variation in age of onset and the occurrence of latency and relapses require, for their explanation, an endogenous underlying risk factor essential for the pathogenesis and clinical manifestation of the disease. In this paper we propose one such putative factor, a ‘melanoma-like neuromelanin’ (MLN), the involvement of which is supported by the available epidemiological data, and we discuss the relevance of this factor to the interpretation of past and current studies on MS and on the possible direction of future studies. The many factors postulated to play a role in the aetiology of MS are summarised in Table 1, according to the strength of the evidence. The candidate factors with possible relevance in MS will be considered only in relation to MLN. Alternative and/or additional influences of these factors [3] are included in Table 1 but are largely beyond the scope of this debate.

**Table 1:** Environmental and genetic risk factors for multiple sclerosis

**MS risk factor**	Risk*		**Observations**	**Postulated mechanism**	**Alternative mechanism**
				**1. Compromise of MS**-**protective immune reactions favouring the biosynthesis of MLN**	
Human endogenous retrovirus-W (HERV-W)	^ X^	F	HERV-W and/or syncytin-1 more frequently detectable/elevated in MS, increased levels of antibodies against HERV GAG and ENV antigens in MS, related to the activity of disease [4-8]	Overexpression of the syncytin-1 gene encoded by HERV-W disturbs redox regulation in glial cells [9]	
Time in life of infection	E^●●●^	P	Virtually all MS patients experienced previous EBV-infection and had elevated levels of anti- EBV-EBNA1 antibody in comparison with control groups [3,10,11]. Previous EBV-infection years or decade(s) before onset of MS [12]. Expanded T-helper cell populations recognizing an epitope of the EBV antigen EBNA1 in MS patients [13]	Infection with EBV early in life can establish MS protective immunity [3,10,11,14]. Infection with EBV delayed in life after immune response against an epitope nested in FENIAEGLRALLARSHVER (partial sequence of EBNA-1) is primed (see next entry) to form T- helper cells instead of regulatory T-cells essential for MS protection [14]	(Irrespective of time in life): Clonal expansion of lymphocytes in the CNS, or EBV infection triggers autoimmunity via molecular mimicry
with Epstein-Barr virus (EBV)	§				
	E	F			
Involvement of infection with measles, varicella and herpes simplex viruses and with 12 or more other pathogens	E^●●●^	P	Higher antibody-levels against diverse pathogens in particular measles, varicella, herpes simplex viruses, and EBV in MS-patients [10,15,16], intrathecal synthesis of antibodies against these viruses (including as in particular also rubella virus) [17,18]	Immune responses against diverse agents generates MS-protection by cross-reaction of a self-specific CD8^+^-T-cell response against a peptide MPVPSAPSTMPVPSAPST belonging to the human endogenous retrovirus W (HERV-W), encoded on the complementary DNA-strand of the syncytin-1 gene [14,19]	Immunological trigger for inflammatory demyelination
	§				
	§				
Infection with *Chlamydia pneumoniae* and/or human herpes virus-6	E §	F	More frequent detection of genomes of these agents in MS [15,20,21] More frequent IgM-specific antibodies against *Chlamydia pneumoniae* in paediatric onset MS [15]	Persisting infections can prime immune response against an epitope nested in FENIAEGLRALLARSHVER (partial sequence of EBV EBNA-1) to induce T-helper cells [14]	
Worm infestation	E^●●^	P	Worm infestation less frequent in MS and treatment of worm infections leads to relapse of MS [22]	Contributes an immune stimulatory context that favours the generation of regulatory T-cells	
Antihistamines	E ^w^	P	Protective effects of antihistamines in MS [23]	Antihistamines suppress unfavourable allergic reactions competing with 'anti-parasite’-like reactions	
HLA-polymorphism	G	P	Main HLA class I molecule A*0201 for the HLA-A0201 associated with a significantly reduced MS risk (OR = 0.52, *P* = 0.0015) [24]	Ability of HLA-polymorphism for immune presentation of the peptide MPVPSAPSTMPVPSAPST is good such as with HLA-A0201 (frequency of about 30% in a European population) [14]	See the text
Interleukin-2 receptor α (IL-2Rα)	G	P	Mutations in IL-2Rα gene in MS more frequent [25]	Component of the CD-25 molecule of regulatory T-cells, critical involvement of these cells in MS-protection	
Interleukin-7 receptor α (IL-7Rα)	G	P	Mutations in IL-7Rα gene in MS more frequent [26]	Important for maintenance of CD8^+^-T-cell memory, critical is a long-persisting MS-protective cellular immune reaction	
n-3-polyunsaturated fatty acids	E	P	Reduced MS risk with diet rich in n-3 poly-unsaturated fatty acids [27]	Dietary factors leading to an enrichment of ganglioside-content of T-cells. The MS-protective immune reaction seems to be an immune repair mediated by gangliosides [14,19,28,29]	
				**2. Factors favouring the biosynthesis of MLN not predominantly involving the immune system**	
Vitamin D-deficiency	E^●●●^ §	F	Month of birth-effect [30,31]. Low levels of vitamin D in MS patients [32-34]	Deficiency pre-birth and after birth reduces intracellular glutathione [35]	Levels of vitamin D experienced *in utero* can have long-lasting effects on the development of numerous organ systems, including the CNS; during life, vitamin D has clear immunomodulatory functions
Low sun exposure	E^●●^	F	Influence of place of residence, MS risk increases with higher latitude [3,32]	Reduced exposure to sunlight rich in UV-B dependent on geographical latitude [3,32]	
Selenium deficiency	E ^w^	F	MS more frequent in regions with low levels of selenium in soil [36,37]	Selenium deficiency reduces levels of the seleno-enzyme glutathione-peroxidase [38]	
Female sex hormones	E §	F	Dependence of MS risk on gender. MS risk in young children indepedent from gender but increased girl/boy rate in puberty [31,32]. Reduced MS risk in pregnancy, elevated MS risk after pregnancy and after the menopause [39]	Syncytin-1 gene has a sensitivity for female sex hormones, (gene product has physiological role in placenta) [39]	Altered antigen reactivity, tolerance, epigenetic effects
Smoking	E^●●^	F	Higher risk of MS in cigarette smokers [40]	Nicotine accumulates in melanin containing cells and interferes with melanin synthesis [41]	Nitric oxide-mediated demyelination, axonal loss and epigenetic effects
Iron-load	E	F	MS association to eating of meat [27]. Iron accumulation early in MS plaques [42,43]	Melanoma-melanin is incorporating iron [44-48]	
				**3. Oxidative charging of MLN**	
Vitamin D-deficiency	E^●●●^	F	See above	See above, glutathione is needed for discharging of MLN	
	§				
Low sun exposure	E^●●^	F	See above	See above, glutathione is needed for discharging of MLN	
Selenium deficiency	E ^w^	F	See above	See above, glutathione-peroxidase is needed for discharging of MLN Iron containing MLN is charged by ionizing radiation/ cosmic radiation [49]. Cosmic radiation	
Geomagnetic disturbances/ Cosmic radiation	E^●●^	F	MS relates to geomagnetic 60° latitude [1]		
	E	F	MS association to mountain regions [50]		
				**4. Physiological influence on activity of neuromelanin**	
Visible light	E §	P	Increased risk of MS onset (1st attack) in the lightest months of the year [31,51]	The hormone melatonin regulates the daily activity of neuromelanins (light-triggered day-night rhythm)	
				**5. Formation of short living reactive oxygen species and radicals (ROS) by mitochondria**	
Psycho-physical trauma	E	F	Increased MS risk in relations to psycho-physical trauma, detection of ROS-related products in MS [52]	Traumatic events lead to the generation of ROS by mitochondria	Dysregulation of the hypothalamic-pituary-adrenal axis
Heat/fever	E	F	Heat as attack provoking factor in MS [53,54]	Heat leads to the generation of ROS by mitochondria	
	E	F	MS protective effect of the introduction of anti-pyretics [53] and of the antibiotic penicillin [55]	Fever leads to the generation of ROS by mitochondria	
Chronic stress reaction	E ^w^	F	Decreased ubiquinone, and increased endogenous digoxin and metabolites of oxidative stress in MS [56]	Over-activation of the cellular mevalonate pathway with decrease of ubiquinone, and increase of endogenous digoxin and ROS production by mitochondria [56]	As above
				**6. Formation of long**-**living reactive oxygen species and radicals (ROS) requiring typically nitrogen oxide as a co**-**substrate**	
Infection/Inflammation	E	F	A small blood vessel is often running through the plaque, the end stage of MS pathology [57]	Adherent polymorph nuclear cells in small blood vessels form nitrogen oxide (NO) that is not readily cleared	
Gout	E	P	MS is extremely rare in patients with gout and levels of uric acid are about 10 to 15% lower in patients with MS [58]	Radical scavenger function of uric acid for nitrogen-containing ROS [58]	
decreased uric acid	E	F			
Glatiramer-acetate	E	P	An agent with therapeutic benefit in MS	Inhibition of NO synthase of mononuclear cells [59]	

Categorization of risks (see footnotes), observations, postulated mechanisms with respect to a hypothetical melanoma-like neuromelanin (MLN) ordered in groups of 6 aspects, and alternative mechanisms as suggested in ref [32].

* E = environmental risk, G = genetic risk, ^X^ = genetic factor but indirectly environmental risk; P = protective, F = fatal; ^●●●^ = very strong evidence; ^●●^ = strong evidence; ^w^ = evidence weak. § In a group of German patients with onset of MS in childhood, studied by Hanefeld and co-workers, diverse observations were made more or less in the same study group. Categorization for the evidence of these observations is based on comparison of p-values by multiple testing with Bonferroni-correction as described in ref. [15].

It is, however, important to note that the quality of the evidence for the relevance of the many described factors to the pathogenesis and clinical outcome of MS varies considerably. To guide the reader, we make clear in the Table 1 those observations that are supported by firm evidence. All interpretations in the text beyond the section ‘Evidence for concomitant effects’ are only speculative but, again, those considered to be of key relevance for the proposed unifying concept are likewise highlighted (●●● = very relevant and ●● = relevant).

### Evidence for concomitant effects

An illustrative group of paediatric patients studied by Hanefeld and co-workers [10,15,17,18] emphasises the wide range of factors of putative relevance in the pathogenesis of MS (Table 1, footnote §). The most notable of these are evidence of past infection with Epstein-Barr virus (EBV) in virtually all patients [10,60] ●●●, and altered immune responses to infections with several other viruses, in particular with measles, varicella, and herpes simplex viruses [15] ●●●, and with *Chlamydia pneumoniae*[15,20]. Moreover, production of specific antibody against measles, rubella, varicella, EBV and herpes simplex viruses in the brain/cerebrospinal fluid is also a hallmark of MS [17,18] ●●. Further observations reveal an association of MS risk with the season of birth, postulated to be related to low vitamin D levels experienced *in utero* (pre-birth) [19,30], exposure to visible light close to the time of onset of the disease [31,51] and, finally, a dependence of MS risk on female sex hormones that commences only in late childhood, becoming more pronounced in adolescence, and reaching maximal values in adults: with the girl:boy ratio on age at onset in young children (< 8 years) being 1.2:1, compared with 3:1 in adults [31,32]. The described risk factors for MS, observed in parallel and numbering around 16, are listed in Table 1, footnote §.

### Is a melanoma-like neuromelanin (MLN) the ‘missing link’?

Current explanations as to how the diverse risk factors might cooperate to cause MS [3], fail to delineate any single candidate factor able to explain concomitance of factors operating at widely differing points or periods of time in life. There is, however, one – though still hypothetical – factor that is in principle able to form the basis of such time-spanning concomitance and we have already speculated that this is the ‘missing link’; namely, a melanoma-like neuromelanin (MLN) [14], and this is supported by the fact that the initiating steps in MS as well as in melanoma [61] occur in cells derived from the neural crest. This encouraged us to seek for parallels in the aetiology of the two diseases, parallels that might be ascribed to the biosynthesis and action of a hypothetical melanoma-like neuromelanin. In both diseases various environmental causative agents have been implicated in pathogenesis and some may combine, perhaps synergistically, to produce the respective disease. These include an overexpression of genes coding for the envelope proteins of human endogenous retroviruses (HERVs), of group W in MS [4-8] and of K in melanoma [62], although the increased amount of the HERV-W ENV protein (syncytin-1) in nerve tissue is neither a genetically determined risk nor a result of an infectious behavior of these elements with a viral ancestry, encoded by the human genome.

The importance of the establishment of protective immunoregulatory surveillance networks for the prevention of MS is emphasised by the so-called hygiene hypothesis. There is growing evidence from many sources that environmental factors in the industrialised nations that isolate the human population from contact with exogenous viral, microbial and parasitic stimuli that millions of years of evolution have led the immune system to ‘expect’ has led to a failure in the development of immunoregulatory networks and to an increase in incidence of several classes of disease characterised by chronic inflammation and autoimmune phenomena [61,63]. With particular relevance to MS, exposure to certain pathogens such as the Epstein-Barr virus which once regularly occurred in infancy now often occurs only years later [10,12].

In the following sections we discuss the possible aetiological role of the hypothetical MLN, drawing parallels with melanoma, and the putative role of HERVs, Epstein-Barr virus, other infections and other non-infectious environmental factors [3,33] that could affect the biosynthesis of MLN, possibly commencing even before birth. We also discuss its subsequent oxidative charging and discharging and its relationship to other described co-factors acting at quite different times in life that might also be involved throughout the pathological processes that finally determine the clinical manifestations of MS.

### Characterization of MLN

Neuromelanins behave as quasi-polymeric redox-pigments [42]. Relatively large quantities are necessary for chemical analysis [43], and studies on small quantities of the various types of pigments have not, to our knowledge, been undertaken. Since, however, pathological forms of eumelanins of the skin, melanoma-like melanins, incorporate increased amounts of iron and other metals [44-48,64], it is likely that the presence of the hypothetical MLN would be detectable by determining iron levels in the relevant tissues ●●. Iron has been detected in MS plaques and it has been shown by histopathology, three-dimensional enhanced T2*-weighted angiography, and other forms of nuclear magnetic resonance applicable to living persons, that iron is already present in the earliest stages of demyelinating disease, although iron levels showed no correlation with time elapsed since the initial clinical event nor with the degree of disability [65,66].

Melanins, having diverse functions, are not primarily involved in the metabolism of reactive oxygen species and radicals (ROS) [45] since the membranes of the two organelles, mitochondria and melanosomes, are barriers for the migration at least of the negatively charged ROS-anions. Although evidence for the existence of an equivalent of melanosome membranes in the brain is still missing [67,68], it seems highly probable that the contact between a substantial amount of ROS and melanins results not only from an over-production and/or a failure of the destruction of ROS in the mitochondria but also when there is leakage of membranes that can be induced by the ROS themselves.

Melanins have the potential to act as quasi catalysts, becoming more oxidatively charged with every redox-reaction they participate in, so that they depend on systems for their subsequent reduction [45]. While the normal physiological forms of these pigments transform ROS to harmless oxygen species, abnormal oxidatively charged forms can generate harmful longer living species, in particular peroxynitrite ●●●.

Pathological forms of melanin, as encountered in melanomas, incorporate more iron [44-48], and are thus enabled to interact more profoundly with radiation. Whereas visible and ultraviolet light is transformed to heat (photon/phonon coupling), higher energy photons can cause photoionization which is dependent on the Fenton reaction of iron, Table 2[69] ●●●. Upon dissipation of the absorbed energy the pigment becomes electrically and oxidatively charged [49] ●●.

**Table 2:** Five chemical reactions with supposed critical relevance in relation to MLN and MS

1	Fenton reaction of iron: (1) Fe^2+^ + H_2_O_2_ → Fe^3+^ + HO• + OH^−^ and (2) Fe^3+^ + H_2_O_2_ → Fe^2+^ + HOO• + H^+^
	This reaction can become induced by ionizing radiation/ geomagnetic disturbances and
	is involved in oxidative charging of MLN (A → B in Figure 1) [49,69]. ^P^
2	Vitamin D-dependent γ-glutamyl-transpeptidase reaction generating glutathione in astrocytes, allowing for discharging of MLN (B → A in Figure 1) [35]. ^P^
3	Radical scavenging of nitric oxide by means of uric acid [58]. ^P^
4	Generation of peroxynitrite by a quasi-catalytical reaction of oxidatively charged MLN [45]. ^F^
5	Demyelination by means of reaction of peroxynitrite with myelin [70]. ^F^

^P^ = MS protective influence, and ^F^ = fatal influence.

Melanins can also be extremely long-lived [45] and this could, in principle, explain the concomitance of many factors in MS that operate over a wide range of time, from before birth and through early and later childhood until the onset of MS, typically in young adults.

### Environmental influences linked to the biogenesis and behavior of MLN

**Figure 1:**
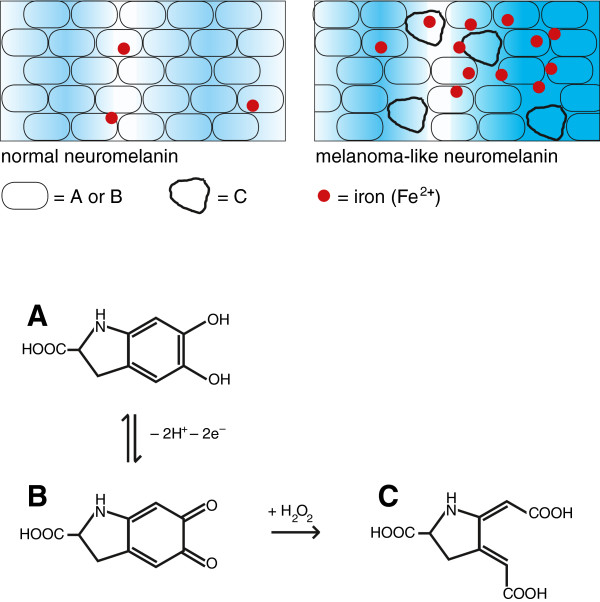
**Schematic description of normal neuromelanin and melanoma**-**like neuromelanin (MLN).** Partial structures of the subunits **A**: 5,6-dihydroxyindole-2-carboxylic acid, **B**: the oxidised *ortho*-quinone form, and **C**: an initial product formed upon reaction of B with H_2_O_2_; 'red point symbol': iron (Fe^2+^). MLN has a greater tendency to become oxidatively charged (increased B/A-ratio) indicated in the background by stronger blue shading.

The critical point in the biogenesis of MLN seems to be a high susceptibility to peroxides of the carbon-carbon bond between the two carbonyl-groups of the (hypothetical) *ortho*-quinone structure in the melanin subunits [45] and its precursors ●●● (Figure 1). Glutathione peroxidase as well as glutathione are needed to destroy all peroxides and a shortage of the enzyme is related to redox processes disturbed by syncytin-1 [9,14].

Whereas the physiological neuromelanin pigment oscillates between the oxidised and reduced state, a hypothetical pathological form able to accumulate iron would have a greater amplitude of charging and a tendency to become pro-oxidatively charged (Figure 1). An oscillating process of oxidative charging and discharging of neuromelanin appears to be promoted by the combination of two equally important environmental effects, charging triggered by geomagnetic disturbances/cosmic radiation [1] and discharging by reduction equivalents that must be generated by the cells in a vitamin D-dependent manner [35] ●●●.

The possible importance of geomagnetic disturbances/cosmic radiation is supported by a recent meta-analysis that showed a significantly closer association of MS to 60° magnetic latitude as compared with 60° geographic latitude [1] ●●. One consequence of this important study is that cosmic radiation on Earth’s surface, although of low intensity (about 0.1 r/year) cannot be described as biologically completely harmless. Another consequence of this study is that vitamin D can no longer be considered as the predominant MS risk factor [3,33], although it must still be considered to be one of the several factors involved. Indeed this vitamin is likely to play a key role in MS as it is involved in the generation of reduction equivalents (glutathione) in the nerve cell, via γ-glutamyl-transpeptidase [35]. Crucially, the oxidative charging and discharging of MLN is an oscillating process, characteristic of a Belousov-Zhabotinsky reaction [71] that could logically explain the spatial and temporal manifestations of MS with plaques, attacks and remissions [19].

We postulate that a pro-oxidatively charged form of an altered melanin, namely MLN, is unable to destroy radicals and reactive oxygen species (ROS) released during abnormal mitochondrial activity (see below) but, instead, transforms them to more harmful long-living species, a process requiring nitrogen oxide (NO) as a co-substrate. Once formed, these abnormal longer-living species are responsible for demyelination in MS [70] ●●●.

### Underlying virological factors in the biogenesis of MLN

It is highly probable that the biogenesis of a pathologic neuromelanin depends on a complex background that compromises the immunological control of the expression of an endogenous retroviral gene product, syncytin-1 [14], as an overexpression of this cellular component can disturb redox processes within the cell [9] and thus favour the biosynthesis of a melanoma-like neuromelanin (MLN). This could prove to be the link between MLN and the characteristic patterns of infections and associated T cell activity that we have described in our previous publications [14,19]. The available evidence points strongly to the root cause of MS being the overexpression of human endogenous retrovirus W (HERV-W) ENV coding for the syncytin-1 protein consequent to a complex immunological background centered on a past infection with Epstein-Barr virus (EBV) 005B [11,14] ●●●. In this context the ‘biography’ of the immune system [14] is of key relevance as an infection with EBV can compromise a MS-protective long-lasting cellular immune surveillance only after the immune response has been primed by other, prior, infections, in particular with *Chlamydia pneumoniae*[10,14,60] and human herpes virus 6 [21].

As a consequence, a MS-protective immune response can become compromised ●●●. The specific candidate targets of the involved regulatory T-cells (T_reg_s) and CD8^+^-T-cells have been delineated [14], see also Table 1. T_reg_s of critical importance for control of HERV-W ENV expression become replaced by T-helper cells with similar specificity ●●●. The generation and maintenance of the postulated HERV-W ENV suppressive and, as a consequence MS-protective, immune surveillance on the one hand and the replacement of T_reg_s by T-helper cells on the other will inevitably have a range of observable effects on the immune network and, thereby, on the overall pattern of immune reactivity. These effects include an epitope spreading of the T-helper cell epitope in EBV EBNA1 [13], an elevated antibody level against EBNA1 [11], an attrition of the CD8^+^ T-cells with specificity for EBV-infected cells though not a reduction of the total number of CD8^+^ T-cells with specificity for EBV [72], and an elevated antibody production against a diverse range of pathogens [15,16]. Those pathogens which appear most strongly associated with the pathogenesis of MS bear the identified candidate target sequences for the T_reg_s as well as for the CD8^+^ effector T-cells on the same protein; namely, measles, varicella, herpes simplex and rubella viruses, the latter only when infection involves the brain, where processing of the viral precursor protein is incomplete. All these exogenous viruses support, by their involvement in the immune network, a MS-protective cellular immune response that becomes compromised before the clinical onset of MS. Again, light is shed on the identity of the candidate epitope of relevance for the MS-protective CD8^+^ T-cells by the postulated analogy to melanoma. In both cases it is a peptide coded for by an additional open reading frame in the gene complex coding for the human endogenous retroviral ENV protein [14,73]. The candidate peptide of putative relevance for MS is, surprisingly, coded on the counter-strand of the ENV-coding DNA strand [14]. The candidate mechanism suppressing the activity of HERV-W ENV but inducing a lytic cycle of EBV [28,29,73] seems to operate by causing a shedding of gangliosides of the neo-lacto series to the target cell [28,29].

### MLN might explain the relevant mode of action of diverse factors in the aetiology of MS

In respect to these infective, immunological and other potential MS risk factors, centering on epidemiological observations, a number of questions relevant to the MLN-based concept are raised: Could worm infestation, which has recently been shown to have beneficial effects in MS, be exerting a non-specific immune modulating effect favouring expansion of sub-populations of T_reg_s [22], with allergic immune reactions being counter-productive [23] ●●●? Is a critical involvement of T_reg_s evidenced by the association of MS to mutations in the IL-7Rα gene (CD25 molecule of T_reg_s) [26], and the importance of a long-lasting CD8^+^T- cell response by mutations in the IL-2Rα gene [25]? Moreover, could it be that the set of HLA-antigens of the patients [74] is less able to present the peptides that have been delineated and incriminated as essential for the relevant T_reg_s and effector T-cells of the suggested MS protective immune response [14] ●●●? Could it be that the suggested effector mechanism, a ganglioside-mediated suppression of HERV gene expression [14,19], is reflected in a seemingly protective effect of dietary n-3 polyunsaturated linoic acids that enhance the ganglioside content of T-cells [27]?

Other questions arise concerning additional factors that do not predominantly involve the immune system. Thus, could vitamin D deficiency [3,33,34] as well as a low exposure to bright sunlight rich in UV-B resulting in low intracellular glutathione [35] favour the biogenesis of MLN and play a role in a compromised ability to reduce oxidatively charged MLN ●●●? Could a selenium deficiency, another factor linked epidemiologically with a risk of MS [36,37], result in reduced levels of glutathione peroxidase (a selenium-containing enzyme) [38]? Could smoking [40] lead to enrichment of nicotine in melanin containing cells and thus to a disturbance of melanin biosynthesis [41] ●●? Could the risk-enhancing influence of female sex hormones be due to a steroid sensitivity of the endogenous retroviral syncytin gene that has a physiological role in the placenta [75] ●●? Moreover, could the hormone melatonin that regulates melanin activity explain a higher rate of MS onset and of attacks in the lighter months of the year [31,32]?

Could psycho-physical trauma act via mitochondrial production of ROS [52]? Could a reduction of the risk of MS in some regions in birth cohorts since the 1920′s be attributable to the introduction of anti-pyretics [53] and, some time later, of antibiotics [55] with their influence on pyogenic infections, since reactive oxygen species and radicals (ROS) are released from mitochondria under the influence of the unphysiological conditions of heat and fever [54] ●●? Could it be also that the bulk of NO in the process finally manifesting as MS is produced by peripheral blood adherent mononuclear cells in small blood vessels that can still be seen in the plaques as the pathological end stages of MS [57] ●●? The more harmful nitrogen-containing ROS such as peroxynitrite, able to cause demyelination, can, however, be scavenged by uric acid [58] ●●. Interestingly, in this context, MS is extremely rare in patients with gout and the serum concentration of uric acid tends to be about 10-15% lower in MS patients than in controls [58]. Could a currently used MS remedy, glatiramer acetate, work predominantly as an inhibitor of NO synthases [59]? Finally, light might be shed on what is perhaps the least understood aspect of MS, the question of what is going wrong with the mitochondria and why they have a higher tendency to produce ROS? It has been postulated that there is an activation of the cellular mevalonate pathway in MS with production of endogenous digoxin and a decrease of ubiquitin levels that impairs mitochondrial function and contributes to oxidative stress [56]. The question is raised whether this aberrant mitochondrial function might be caused by abnormal expression and activity of HERVs [76].

## Discussion

The melanin-based concept is a promising and unifying one that could explain several of the enigmas of MS, including the distribution of age at onset since there is only a very small amount of neuromelanin in childhood, in particular in early childhood [45], and also the complex basis of latency and the Belousov-Zhabotinsky characteristic of the spatial and temporal characteristics of the disease (plaques, attacks, remissions) [19,71]. Although many putative factors appear to be involved at the various states of the pathogenesis and clinical manifestation of MS, the generation and maintenance of MLN is, if the hypothesis is correct, likely to be a key feature throughout. Moreover, the concept sheds light, in principle, on a number of epidemiological observations on factors possibly involved in the aetiology of MS, in particular EBV infection, the HLA system, geomagnetic disturbances/cosmic radiation, vitamin D levels, and smoking, as well as several others listed in Table 1. The possible involvement of cosmic radiation [77,78] is an aspect in which interest has been revived [1] and it has been investigated recently in a mouse model of Alzheimer’s disease [79]. Although definitive answers cannot be given to any of the many specific questions raised on possible relations to the hypothetical MLN, it should be noted that the concept as a whole is unique amongst the hypotheses on MS in not contradicting the birth cohort trends [2]. Also, as the many factors referred to above vary from region to region and from patient to patient, the involvement of MLN could be a constant feature despite the heterogeneous nature of the disease.

The concept discussed in this debate paper differs markedly from competing hypotheses and from current concepts that are focused on cell-mediated immune reactions directed against myelin. In this context, infiltrating lymphocytes are typically absent in progressing and expanding MS lesions, and there is a prominent oligodendrocyte loss and apoptosis, supporting the concept that the plaque formation has indeed some basis other than cell-mediated immunity against a myelin or oligodendrocyte antigen [80,81]. From this concept, and from the conclusions of other workers [82], it now appears that autoimmune reactions in MS are only secondary phenomena.

The unifying concept has many elements that may vary in their impact from patient to patient. Though less so for the unifying concept itself, this may be of relevance more for secondary phenomena, such as autoimmune reactions and phenomena associated with the compartmentalisation of iron. Thus the accumulation of iron in MLN may result in a shortage of iron in myelin, which could impair the function of nerve cells [83]. These effects might contribute to the heterogeneity of the clinical manifestations of MS ●●.

There is certainly a need to study all the diverse aspects of MS in relation to MLN and iron, including the impact of pregnancy [39] and geological terrain of residence [50]. Unfortunately, neuromelanin is not a molecule that is easily studied [43], especially as there is little or no neuromelanin (the precursor of MLN) in the animals used for experimental models in MS [45] ●●●. Thus, perhaps, emphasis must be placed on the study on iron in the human brain [84] ●●. While prevention of MS may ultimately depend on the development of an effective vaccine against the Epstein-Barr virus that can be administered in early childhood, as the *sine qua non* for the disease is Epstein-Barr virus infection [14,72] ●●●, therapy of established MS poses a greater challenge. Addressing this challenge would be greatly facilitated if there is indeed a unifying factor underlying the complexity of the pathogenesis of MS and we open for debate the possibility that this unifying factor is the generation of a melanoma-like neuromelanin.

## Summary

The concept of an abnormal MLN explains many of the characteristics of MS and we postulate that it is an essential feature of this disease. There are many outstanding unanswered questions surrounding the various pathological, immunological and clinical aspects of MS but a consideration of the underlying role of MLN and the processes leading to its generation and maintenance may help to answer these questions and pave the way to a unified concept of the pathogenesis of this complex disease.

## Abbreviations

DNA: Deoxyribonucleic acid; EBV: Epstein-Barr virus; ENV: Envelope protein; HERV: Human endogenous retrovirus; MLN: Melanoma-like neuromelanin; MS: Multiple sclerosis; NO: Nitrogen oxide; ROS: Reactive oxygen species and radicals; Tregs: Regulatory T-cells.

## Competing interests

The authors declare that they have no competing interests.

## Authors’ contributions

BK and JMG contributed equally. Both authors read and approved the final manuscript.

## Authors’ information

BK, PhD, MD, worked for more than 7 years in the field of biomolecular chemistry on redox pigments of the quinone type and for 22 years in the field of virology. Together with JMG he investigated the possible infectious background of multiple sclerosis and of melanoma, the latter within the frame of the FEBIM studies. JMG, MD, MSc, has worked extensively on the microbiology and immunology of chronic infection, especially tuberculosis, and on the immunology and immunotherapy of cancer.

## Pre-publication history

The pre-publication history for this paper can be accessed here:

http://www.biomedcentral.com/1471-2377/13/91/prepub
